# Supplementary material for: Engineering atomic-scale magnetic fields by dysprosium single atom magnets
Source: Nat Commun. 2021 Jul 7;12:4179. doi: 10.1038/s41467-021-24465-2 (PMC8263604; doi:10.1038/s41467-021-24465-2)
Supplement: Supplementary file 1 — Supplementary Information [file 41467_2021_24465_MOESM1_ESM.pdf]

Supplementary Information

Engineering atomic-scale magnetic fields by dysprosium single atom magnets

A.Singha[1,2,3,†,*], P.Willke[1,2,4,†], T. Bilgeri[5,†], X. Zhang[1,2], H. Brune[5], F. Donati[1,6], A. J. Heinrich[1,6,*], & T. Choi[1,6,*]

[1]Center for Quantum Nanoscience, Institute for Basic Science (IBS), Seoul 03760, Republic of Korea

[2]Ewha Womans University, Seoul 03760, Republic of Korea

[3]Max Planck Institute for Solid State Research, Stuttgart, Germany

[4]Physikalisches Institut, Karlsruhe Institute of Technology, 76131 Karlsruhe, Germany

[5]Institute of Physics, École Polytechnique Fédérale de Lausanne, Station 3, CH-1015 Lausanne, Switzerland

Department of Physics, Ewha Womans University, Seoul 03760, Republic of Korea

1. Analysis of two-state switching

In Fig. S1a, we show time traces of the change in topographic height Δz for different tunnel voltages, taken atop a Dysprosium (Dy) atom with a spin-polarized tip (SP-tip) at a magnetic field of $5\ \mathrm{T}$. The traces reveal switching between two states that stem from different alignments of the Dy spin with respect to the magnetic field of the tip, as already shown in Fig. 1B in the main text. Moreover, the switching rate Γ increases with increasing bias voltage, indicating that

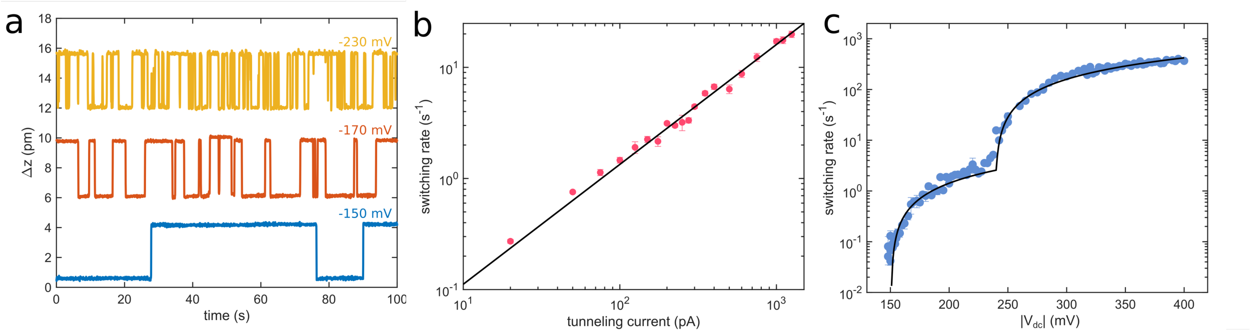

Figure S1 | Magnetic switching of Dy. a, Change in tip height Δz due to the change in magnetic state recorded atop a Dy atom ($T = 1.8\ \mathrm{K}$, $B_z = 5\ \mathrm{T}$, $I = 1.5\ \mathrm{nA}$). The switching rate increases as a function of bias voltage. A linear background due to thermal drift of the z-piezo was subtracted. **b,** Switching rate Γ as a function of tunneling current I. Black line is a fit to the data as described in the text ($T = 1.8\ \mathrm{K}$, $B_z = 5\ \mathrm{T}$, $V_{\mathrm{dc}} = -230\ \mathrm{mV}$). **c,** Switching rate Γ as a function of bias voltage V_{dc} ($T = 1.8\ \mathrm{K}$, $B_z = 5\ \mathrm{T}$). In order to observe the switching rate over 3-4 orders of magnitude, we took data at $1.5\ \mathrm{nA}$ ($150 - 250\ \mathrm{mV}$), $0.15\ \mathrm{nA}$ ($250 - 320\ \mathrm{mV}$) and $0.015\ \mathrm{nA}$ ($315 - 400\ \mathrm{mV}$) and multiplied them by 1, 10, and 100, respectively. Black line is a fit to the model described in the text.

higher energy electrons facilitate switching of the Dy spin state. In order to further characterize the switching rate Γ, we performed measurements as a function of tunneling current I (Fig. S1b) and bias voltage V_{dc} (Fig. S1c). Here, the switching rate contains the total number of switches, both from the up to the down state and vice versa. We fit the tunneling current-dependent switching rate to a model of the form $\Gamma = a \cdot (I/I_0)^N$, which has been used previously to describe random telegraph signal from two-state magnetic switching events in atomic structures[1,2,3]. Here, $I_0 = 1\ \mathrm{pA}$, a is the switching coefficient and N is an exponent. In Table S1 we show the values for a and N obtained from fits to the data in Fig. S1b. In addition, we took data at opposite bias voltage and without magnetic field. All measurements yield values for the exponent $N{\sim}1$, indicating that a single-electron processes is governing the switching.

	$\mathbf{5\ T\ (-230\ mV)}$	$\mathbf{5\ T\ (+230\ mV)}$	$\mathbf{0\ T\ (+230\ mV)}$
$\boldsymbol{a}\ (\boldsymbol{s}^{-1})$	$(9 \pm 4) \cdot 10^{-3}$	$(14 \pm 12) \cdot 10^{-3}$	$(1.2 \pm 1.4) \cdot 10^{-3}$
$\boldsymbol{N}$	1.08 ± 0.07	1.17 ± 0.12	1.11 ± 0.17

Table S1 | Evaluation of current-dependent two-state noise measurements

The voltage-dependent measurements (Fig. S1c), which are also shown in Fig. 1c of the main text, reveal two prominent rate-increasing thresholds. Here, we model them by a piecewise fit to[1] $\Gamma = \sum_{i=1}^{2} H(V - V_i) \cdot c_i \cdot (V - V_i)/V_i$, where $H(V - V_i)$ is the Heaviside step function. We find $|V_1| = (155 \pm 3)\ \mathrm{mV}$ and $|V_2| = (235 \pm 1)\ \mathrm{mV}$. These thresholds lie much higher than the respective transitions for Holmium ($V_1 = 73\ \mathrm{mV}$, $V_2 = 104\ \mathrm{mV}$ and $V_3 = 119\ \mathrm{mV}$). Table S2 contains the evaluation of the fits to c_i and V_i under varying magnetic field and bias polarity as all already used for the current-dependent data (Table S1). All of them yield comparable values for the thresholds (V_1 and V_2). In the limit of large voltages, we obtain a scattering probability of around $\sim (c_2 \cdot e)/I \approx 10^{-7}$ per tunneling electron, which decreases to $\sim 10^{-8}$ for the $0\ \mathrm{T}$ measurement. This low probability is comparable to that obtained for Ho atoms[1].

	$5\ T\ (+1.5\ nA)$	$5\ T\ (-1.5\ nA)$	$0\ T\ (+1.5\ nA)$
$c_1\ (s^{-1})$	7.5 ± 0.7	4.3 ± 0.5	3.4 ± 0.5
$c_2\ (s^{-1})$	1109 ± 41	620 ± 27	172 ± 66
$\|V_1\|\ (mV)$	155 ± 3	151 ± 1	150 ± 2
$\|V_2\|\ (mV)$	235 ± 1	240 ± 2	229 ± 3

Table S2 | Evaluation of voltage-dependent two-state noise measurements.

2. Magnetic tip preparation and characterization

The STM tip used in this work was made of PtIr wire, which was covered with Ag atoms due to indentions into the substrate required for tip shaping. For preparing SP-tips, we picked up Fe atoms from the surface using atomic manipulations[4]. SP-tips with which we performed tip-field sweep ESR measurements, typically contained ~30 Fe atoms at the apex. For most of the tip-field sweep ESR measurements shown in this work, our SP-tips showed magnetic switching on timescales faster than the timescale of our measurements (~100 ms). We note that the magnetic anisotropy energy of our SP-tip which contains nearly 30 Fe atoms, can conservatively be estimated by 30 times the atomic value for bulk Fe of < 2.6 μeV[5,6]. In this case the tip magnetization switches more than once per second at T > 0.045 K. Therefore, under our measurement

conditions the tip should always show magnetic bistability at zero as well as vanishing magnetic fields. Magnetic bistability has been reported for few-atom clusters of Fe on different metal substrates[2,3,7]. It should be noted that such a magnetic bistability is not expected for SP-tips possessing out-of-plane hard axis. This is due to the absence of doubly degenerate magnetic levels in such tips which would result in an almost zero net-magnetization. Moreover, we note that such tips would not be able to efficiently drive ESR transitions in the Fe atom due to the absence of the longitudinal component of the tip-field[8]. As described in the main text, such magnetic bistability of the SP-tip leads to two resonance peaks for Fe atoms in Fe-Dy pairs for all values of external magnetic fields, except at the merging point. However, some SP-tips also showed longer lifetimes of up to several minutes allowing us to probe the resonance associated with the individual tip state.

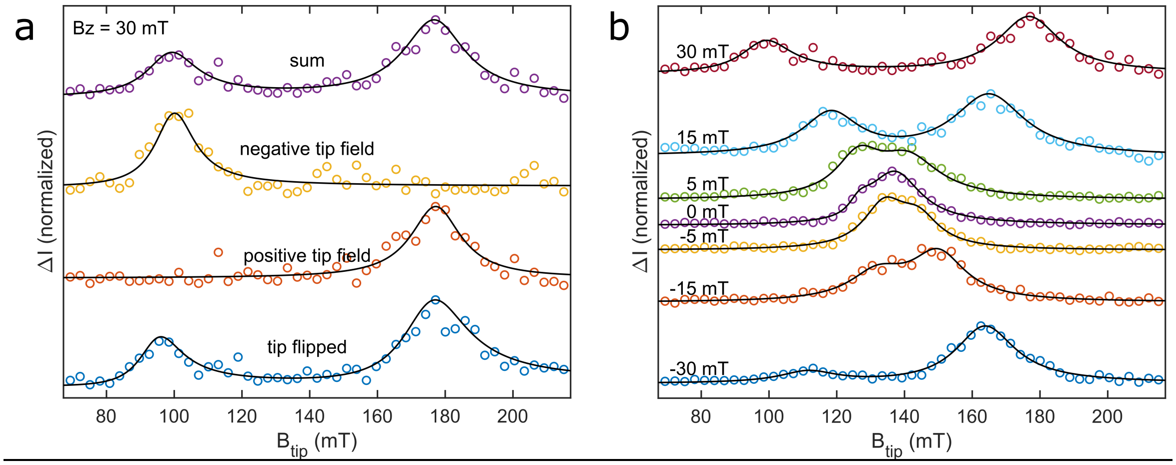

Figure S2 | Tip-field sweep ESR measurements with a slowly switching tip. a, ESR on an Fe-Dy pair at B_{ext} = 30 mT. The curve plotted at positive and negative tip field is the sum of three and two individual traces, respectively. **b,** Full dataset at varying B_Z. All curves are normalized individually. ($T = 1.3$ K, $V_{RF} = 15$ mV, $V_{\mathrm{dc}} = -50$ mV, $f = 21.4$ GHz).

Figure S2 shows ESR measurements of an Fe atom in a Fe-Dy pair with an interatomic distance d = 1.15 nm using a tip that switched only every few minutes. In order to record ESR peaks for both tip states, the traces were measured several times at each magnetic field and subsequently averaged. Exemplary spectra capturing the two different tip states separately are displayed Fig. S2a. By measuring at various external fields (Fig. S2b) we find the same behavior as in datasets in the main text.

From the zero-field splitting we find B_{Dy} = 5.6 ± 2.1 mT in good agreement with the theoretical value B_{Dy} = 6.1 ± 0.2 mT and in line with the data plotted in Fig. 4c.

In order to convert the tip-atom distance to a tip magnetic field, we gauged the tip magnetic fields by acquiring tip-field sweep ESR spectra on a reference Fe atom. This characterization was done for five different radio frequencies and three different out-of-plane external fields $B_{ext} = 0 \text{ and} \pm 30 \text{ mT}$. Figure S3a shows the representative case for $B_{ext} = 0 \text{ mT}$. The ESR peaks appear at larger tunnel currents with increasing fixed resonant frequencies. The peak positions f_0 obtained from the fit to the data are shown in Fig. S3b. f_0 scales in good approximation linearly with tunnel current I_t, which is expected for a tip field dominated by exchange interaction[9]. Thus, we interpolate the data using a linear dependence between the tunnel current at resonance and the fixed radio frequencies at a given B_{ext} as shown in Fig. S3b.

$$B_{tip} = \frac{h(\mathrm{m}*I_t+\mathrm{f}_{offset})}{2\mu_{Fe}} \qquad \text{(eq. S1)}$$

Here, m is the gradient of the fitted curve and f_{offset} is its y-axis intercept. From this linear dependence we extract the tip-fields at any arbitrary tunnel current I_t.

Note that the tip-fields are not influenced by the presence of the Dy atom in any of the engineered Fe-Dy structures. Firstly, this is supported by the excellent agreement between tip-field sweep and frequency sweep ESR, as shown in Fig. 3c of the main text. If the out-of-plane component of the tip-magnetization during a tip-field sweep ESR measurement is modified due to the magnetic field from the Dy atom, systematic errors would occur in the estimation of the magnetic field sensed by the sensor Fe atom. This would result in a strong deviation between the data obtained from tip-field sweeps and those gathered from frequency sweeps, as larger tip-sample distance for the latter leaves the tip-magnetization fully unaffected. In contrast, the measurements shown in Fig. 3c exhibit an excellent agreement between tip-field sweep and frequency sweep ESR carried out with different SP-tips for several Fe-Dy pairs of varying interatomic distances. Moreover, we also find an excellent agreement between tip-field sweep measurements obtained at two different fixed frequencies (13.5 GHz and 16.38 GHz in Fig. 3c). Changes in the set frequency would necessitate measurements at different range of tip-fields which implies different tip-sample

distances. Any spurious effects or systematic error would thus result in disagreements between measurements taken at different values of f_0, which is again contrary to our observations. Furthermore, we also note that the effect of such systematic errors, if any, would also be visible from the B_{Dy} measurements shown in Fig. 4, where both the number as well as the orientation of different Dy atoms were modified for various structures. Consequently, if the Dy dipolar field would affect the tip field significantly, it would affect the determined Dy field, leading to a non-linear behavior in Fig. 4c and Fig. 4d. In contrary, all measurements are in reasonable agreement with the magnetic moment of Dy obtained through two independent routes, i.e., the measurements shown in Fig. 3 c and our multiplet analysis, as shown from the respective linear trends. Given these experimental evidences, we conclude that our tip-field sweep ESR measurements have negligible influence, if any, from the neighboring Dy atom in all engineered structures.

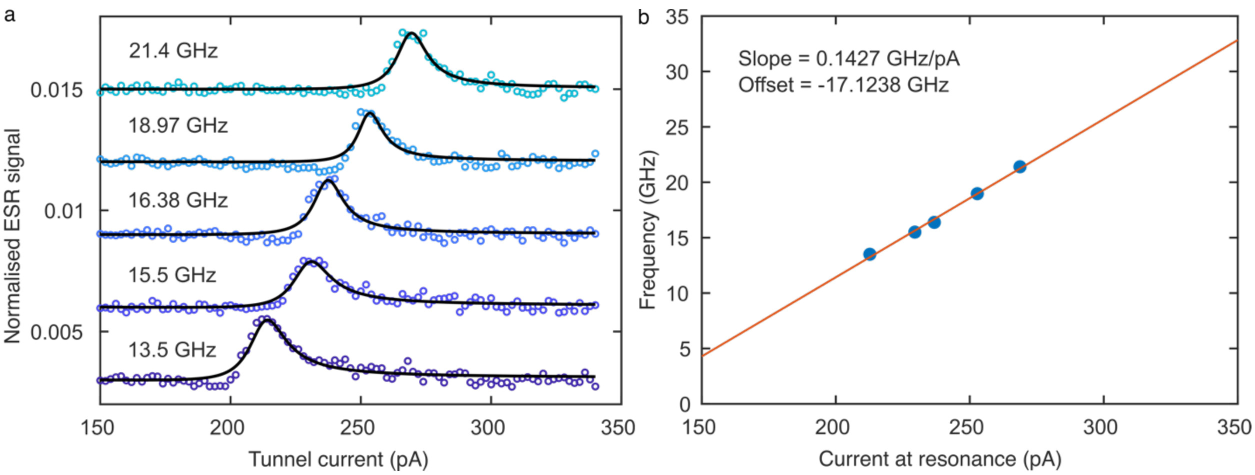

Figure S3 | Characterization of tip-fields. a, Tip-field sweep ESR measurements on a reference isolated Fe atom at five fixed radio frequencies. Solid lines are fits to the data as described in the Methods section ($V_{dc} = -50$ mV, $V_{RF} = 27.5$ mV, $T = 0.6$ K, and $B_{ext} = 0$). **b,** Tip-fields are determined from the slope and offset of the linear dependence extracted from (a) following eq. S1.

3. Vanishing magnetic fields experienced by Dy single atoms

Even in the absence of external magnetic fields (B_{ext} = 0) the Dy atoms are still exposed to several additional sources of local magnetic fields, such as the magnetic field from the SP-tip, the dipolar field originated by any neighboring Fe atom, and the

presence of its own nuclear spin inducing hyperfine coupling. Albeit being small, we discuss in the following their influence in the different types of measurements and note that their influence on the stability can be neglected.

Considering only dipolar coupling between the SP-tip and the surface spins, an upper bound for the magnetic field experienced by the Dy atom during any tip-field sweep ESR can be estimated to be at least a factor of ~5 lower (~20 mT) than that experienced by the Fe atom. However, since the tip-field is likely coupled via exchange interaction to the Fe sensor atom during tip-field sweep ESR (see exponential decay in Fig. S3), which is stronger but decays faster than the dipolar interaction, we would overestimate the tip-field on the Dy atom.

In addition, even after repeated cycles of B_{ext} field ramping through zero within ± 30 mT we did not observe any spin-switching in the Dy atoms. This range exceeds the expected range of all additional field sources listed above (tip-field < ~20 mT, magnetic dipolar coupling of Fe Sensor atom at 1 nm distance of 5 mT, Dy hyperfine splitting < 3 mT [10,11]), so that the Dy spin would already have been swept through its avoided level crossing. Note that all tip-field sweep ESR measurements presented in this work were conducted at fixed values of B_{ext}. For measurements at different values of B_{ext} such as those shown in Fig. 3a, the external magnetic field was ramped at a speed of 10 mT/s. In this regime of magnetic field ramps, we do not expect any Landau Zener tunnelling. During such B_{ext} sweeps, the SP-tip was either retracted or placed far away from any Dy spin. This allowed the Dy atoms to experience the complete absence of any magnetic field during these operations. Despite this, our repeated tip-field sweep ESR measurements on several individual Fe-Dy pair always resulted in an unchanged sign of the dipolar magnetic field from the Dy atoms, indicating the absence of any spin switching in the Dy. Besides these, STM topography scans of Dy atoms performed with magnetic SP-tips, which effectively change the magnitude of the tip-field by the change in proximity of the tip, also did not exhibit any switches in the Dy atoms. This suggests a negligible effect of the magnetic fields from the tip on the Dy spin state.

Given these evidences, we conclude that for $|V_{dc}| < 140$ mV, the magnetic orientation of the Dy atom is always robust, even in the limit of vanishing magnetic fields.

4. Tip-field sweep ESR measurements of an Fe-Fe pair

To further exemplify the zero-field stability of Dy as observed in the tip-field sweep ESR, we here contrast these measurements by a fast-switching spin on a surface, a single Fe atom. In Fig. S4a we show tip-sweep measurements taken on a pair consisting of two Fe atoms 0.81 nm apart. The stray field of a nearby stable magnet such as Dy simply offsets the merging point towards finite positive or negative external field. An unstable nearby magnetic atom with a stray field switching faster than the timescale of our measurements will lead to both types of offsets being present at the same time. Consequently, we find four resonances at large enough fields, three resonances where the merging points would have appeared if the nearby magnet was stable (as in the case of Fe-Dy pairs), and two resonances at zero external magnetic field. Figure S4b shows extracted resonance currents as a function of the external magnetic fields. The solid lines in Fig. S4b are calculated peak positions using equation 1 of the main text. These are in reasonable agreement with our measurements. The deviations from the a strict linearity at larger tip-fields can be attributed to a strong exchange interaction between the two Fe atoms in the pair at such a close distance of 0.81 nm, as also reported in Ref. [12].

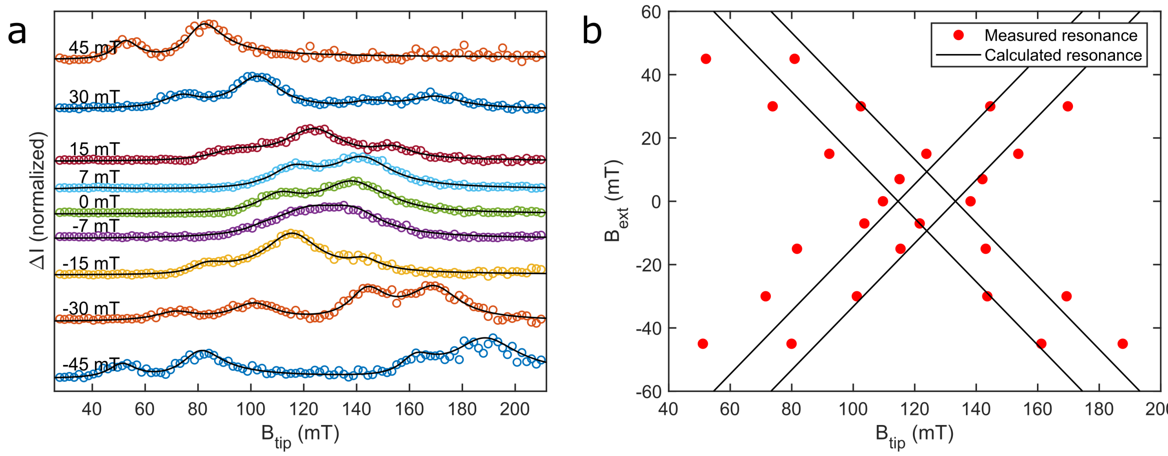

Figure S4 | Low-field tip-sweep ESR measurements of an Fe-Fe pair. a, ESR measurements at different external magnetic fields on an Fe atom in Fe-Fe pair (*d* = 0.81 nm). Solid red curves are fits as described in the Methods. Data for different B_{ext} are vertically offset for clarity ($T = 1.7\,\mathrm{K}$, $V_{RF} = 15\,\mathrm{mV}, V_{dc} = -50\,\mathrm{mV}$, $f = 16.25\,\mathrm{GHz}$). **b,** Measured resonance tip-fields plotted as a function of the

external magnetic field. The calculated values use eq. (1) from the main text and an Fe magnetic moment of 5.44 μ_B.

5. Frequency sweep ESR measurements on Fe-Dy pairs

In addition to tip-field sweep ESR measurements, we performed frequency-sweep ESR on the Fe atoms in the Fe-Dy pairs. This type of measurement has been used more frequently in former works[1,8,13]. Figure S5 presents a series of resonances measured on an Fe sensor atom which is 1.82 nm away from a neighboring Dy atom. In order to demonstrate the effect of the two distinct magnetic orientations of Dy on the Fe sensor, we modified the Dy spin state by using a voltage pulse, $|V_{dc}|$ > 150 mV. This allows us to prepare the Dy atom in the "spin down" configuration, resulting in a higher effective magnetic field experienced by the Fe sensor. Consequently, the ESR peak of Fe shifts to a higher frequency compared to the initial case (lower vs middle spectra). The original peak position can only be restored following another manipulation of the Dy spin state by an additional exposure to high energy ($|V_{dc}|$ > 150 mV) tunneling current atop Dy, thus switching it back to the "spin up" state. These measurements demonstrate again that we can controllably switch the longtime stable magnetic states of the Dy atom. Note that the change in the resonance frequency Δf of the Fe atom during such experiments, is related to the out-of-plane magnetic moments of Fe and Dy atom as [12] :

$$\Delta f = \frac{\mu_0}{\pi\, h\, d^3}\mu_{\mathrm{Dy}}\mu_{\mathrm{Fe}} = \frac{4}{h}B_{\mathrm{Dy}}\,\mu_{\mathrm{Fe}} \qquad \text{(eq. S2)}$$

The second equality in eq. S2 uses the expression for the dipolar magnetic field from the Dy atom: $\mathrm{B}_{\mathrm{Dy}} = \frac{\mu_0\,\mu_{\mathrm{Dy}}}{4\pi\, d^3}$. In order to extract B_{Dy} from the Δf values obtained from all frequency sweep measurements, we use the known value of μ_{Fe} from Ref. [12] in eq. S2, which are shown in Fig 3c of the main text. Note that it is possible to perform such

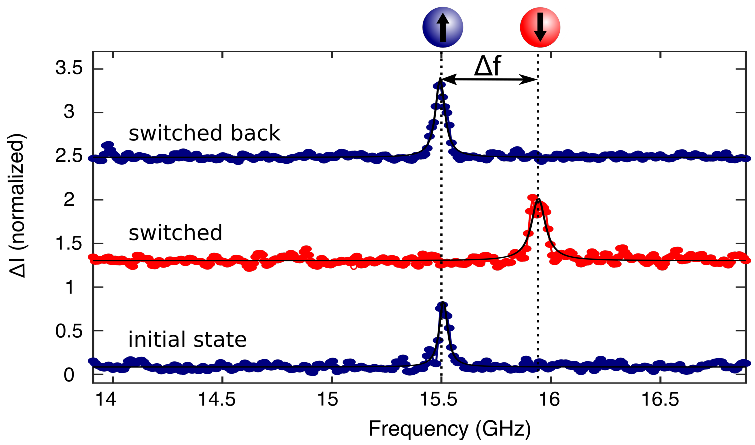

Figure S5 | Frequency sweep ESR measurements on an Fe atom in an Fe-Dy pair. A series of frequency sweep ESR scans on a sensor Fe atom placed at 1.82 nm distance from a target Dy. The lowest spectrum was initially taken on the pair. The middle one was taken after reversing the magnetic state by high energy electrons ($|V_{dc}|$ >150 mV) that correlated with a change in topographic height as indicated in Fig 1b. The upper spectrum was taken after a second change in the magnetic state using the tunnel current (T=1.8 K, B_{ext}^{x} =1.4 T, B_{ext}^{z} = 0.125 T, I_t = 20 pA, V_{dc} = 100 mV, V_{RF} = 15 mV).

frequency-sweep measurements even without any external magnetic field[9], unlike the conventional approach which requires a significantly large external magnetic field. However, in such cases the resonance is driven only by the magnetic field from the tip, which typically results in a broad resonance with linewidths easily exceeding a few GHz, as shown in Ref. [5]. This makes it challenging to infer any small frequency shift in the Fe resonance peak (0.5 GHz for an Fe-Dy pair with d = 1.82 nm) that is expected for any concomitant change of the magnetic orientation of a neighboring Dy atom.

6. Atom manipulations on Dy single atoms

Dy atoms on bilayer MgO were found to adsorb almost exclusively on the bridge sites, that is atop the lattice position between two oxygen atoms of MgO. Thus, the investigation of Dy on top sites as well as the creation of atomically precise structures required the ability to perform single atom manipulation. Dy adatoms could be picked up with almost 100% success rate when approaching the tip towards the Dy adatom under application of a negative bias of a few hundred mV. Dropoff was performed by approaching the tip towards bare MgO under application of negligible bias. Creation of

structures with specific Fe-Dy separations was done by first centering the tip on top of the Fe atom and by subsequently moving it laterally towards the targeted lattice site. Fig. S6 exhibits an example of such pick up and drop off sequences leading to the creation of the Fe-Dy_4 structure described in the main text. Different types of atoms and lattice positions could be distinguished easily by their different apparent topographic height (Fig. S6).

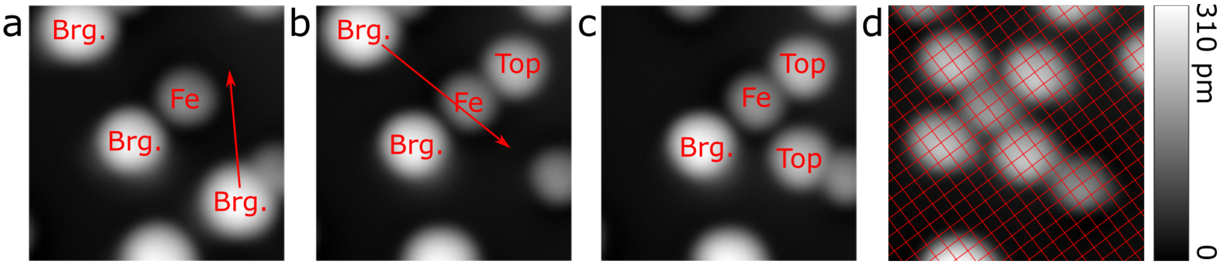

Figure S6 | Atom manipulation sequence. (a) Topographic image showing an Fe atom surrounded by Dy atoms adsorbed on bridge sites (denoted as Brg.) on bilayer MgO. The latter can easily be spotted due to its characteristically large height of around 310 pm. (b,c) The surrounding bridge site atoms are being picked up and moved to O sites at a distance of four lattice sites from the central Fe. Their height is around 230 pm. (d) The finished structure with four top site Dy atoms placed at a distance of four lattice from the central Fe atom. ($T = 2$ K, $V_{dc} = 100$ mV, $I = 20$ pA).

7. Thermal and magnetic stability of Dy single atoms

We find that both magnetic states of Dy single atoms remain thermally stable at least up to 15 K at $B_{ext} = 5$ T. Figure S7a depicts an STM topography showing a chain of 6 Dy atoms that were purposely arranged for testing the thermal stability of their spin states. We detect the Dy spin state by acquiring tunnel current maps of the atoms (Figure S7b). This method is similar to the change in topographic height shown in Fig. 1b in the main text and utilizes the fact that the tunnel current depends on the Dy spin orientation. To conduct these measurements, the feedback loop is opened and the tip is scanned at a fixed height across the sample. Their magnetic states were prepared at 1 K to have '101010' configuration ("42" in binary), where '1' and '0' refer to spin-up and spin-down state of a single Dy atom.

The sample is then heated up to 15 K in two steps (10 K, 15 K). Besides this, a high magnetic field of $B_{ext} = 5\ T$ was applied to additionally facilitate Orbach processes for

the Dy spins. After each intermediate step, the sample is allowed to cool down again to the base temperature of 1-2 K. Subsequently, the magnetic states of the Dy atoms are probed for spin flips by acquiring tunnel current maps as described above (Fig. S7b, second from top to bottom). As evident from the line profiles presented in Fig. S7c, all Dy atoms retain their magnetic orientation. The stability was additionally checked with another 5 Dy atom structure. In both cases no spin flip of any Dy atom was detected up to 15 K at 5 T out-of-plane magnetic field.

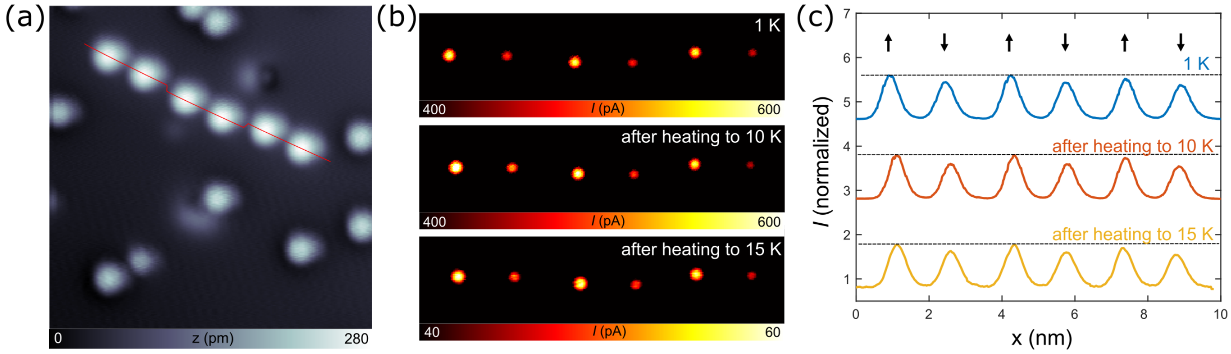

Figure S7 | Thermal stability in Dy single atoms. (a) Topographic image showing a structure of 6 Dy atoms aligned by atom manipulation (T=1.0 K, B_{ext} = 5 T, I_t = 20 pA, V_{dc}= -100 mV). **(b)** A series of constant height STM images of the structure shown in (a). All measurements are performed using an SP-tip at the base temperature of T = 1-2 K, upon sequential heating up to 15 K at $B_{ext} = 5$ T. V_{dc}= 50 mV. **(c)** Line profiles indicate that the prepared magnetic states of the Dy atoms remain intact even after these thermal cycles. Horizontal dotted lines are guides to the eye, showing that the 1st, 3rd, and 5th atom ("spin up") always show larger currents than the 2nd, 4th, and 6th atom ("spin down"), respectively.

8. Multiplet analysis

The spectrum of eigenstates of the Dy atoms has been obtained using the multielectron Quanty code[14]. The Hamiltonian for the 4f electrons

$$\mathcal{H} = \mathcal{H}_{e-e} + \mathcal{H}_{SO} + \mathcal{H}_{CF} + \mathcal{H}_{Zeeman},$$

includes the effects of electron-electron interaction, spin orbit coupling, crystal field and external magnetic field, respectively. In this approach, we neglect the average configuration energy provided by the kinetic energy of the electrons, the hyperfine interaction, as well as the spherical part of the electron-electron interaction, as those

contributions simply produce a constant shift to the eigen energies[15]. The non-spherical part of the electron-electron interaction is treated using the Slater approach. The parameters for the atomic value of the Slater integrals as well as for the spin orbit coupling are obtained from the Cowan's atomic structure code[15] assuming [Xe]$4f^96s^2$ configuration[16]. Values of the Slater integrals have been rescaled to 70% of the free atom values in agreement with previous X-ray spectroscopy data[17]. The first two terms in the Hamiltonian lift the degeneracy of the multielectron states into a series of atomic multiplets characterized by the spin, orbital and total angular momentum quantum numbers S^2, L^2, and J^2, respectively. For Dy in a $4f^9$ configuration, the lowest multiplet is characterized by a $S = 5/2$, $L = 5$, and $J = 15/2$ and multiplicity of $2J + 1 = 16$. The crystal field generated by the adsorption on the four-fold symmetric O site is treated in the Wybourne approach[18], including the Racah tensor $C_m^k(\theta, \varphi)$ allowed by the C_{4v} symmetry:

$$\mathcal{H}_{CF} = A_0^2\, C_0^2(\theta, \varphi) + A_0^4\, C_0^4(\theta, \varphi) + A_4^4\, C_4^4(\theta, \varphi) + A_0^6\, C_0^6(\theta, \varphi) + A_4^6\, C_4^6(\theta, \varphi).$$

Finally, the Zeeman term in the Hamiltonian describing the interaction between the 4f electrons and the external magnetic field B reads:

$$\mathcal{H}_{Zeeman} = \mu_B\, (2\boldsymbol{S} + \boldsymbol{L}) \cdot \boldsymbol{B}$$

For the CF model, the values of the A_m^k coefficients determine the zero-field splitting of the magnetic states. Initial values of these parameters $\tilde{A}_m^k$ have been computed using a point charge electrostatic model (PCEM)[18]. Positions and values of the point charges have been obtained from an initial guess based on a previous work on Gd/MgO/Ag(100)[16]. The distance between the Dy atom and the Oxygen underneath has been increased by 10 pm with respect to Gd to match the experimental level splitting of about 240 meV. Positions and values of the point charges are summarized in Supplementary Table S3.

**Ion**	**Charge**	$\boldsymbol{d}_{\perp}$	$\boldsymbol{d}_{\parallel}$
O (underneath)	-2e	-225 pm	0 pm
Mg (2nd nearest neighbours)	+2e	-270 pm	208 pm

O (3rd nearest neighbours)	-2e	-270 pm	294 pm

Supplementary Table S3 | Charges values and positions used for the PCEM model. Vertical distance ($\boldsymbol{d}_{\perp}$) and projected distance on the plane ($\boldsymbol{d}_{\parallel}$) with respect to the Dy atom position are shown for the surface nearest neighbour up to 3rd order.

Similar to the case of Gd, semi-empirical re-scaling parameters κ_m^k have been included to overcome the well-known limitations of this model. These parameters have been identified by comparison with the level splitting from a benchmark multiplet code (MultiX) with in-built PCEM functionality[19]. The semi-empirical re-scaling parameters κ_m^k , and the computed values for the $\tilde{A}_m^k$ and $A_m^k = \kappa_m^k \tilde{A}_m^k$ coefficients are summarized in Supplementary Table S4.

PCEM	$\tilde{A}_0^2$	$\tilde{A}_0^4$	$\tilde{A}_4^4$	$\tilde{A}_0^6$	$\tilde{A}_4^6$
	349.0 meV	63.3 meV	4.4 meV	15.8 meV	1.4 meV
	κ_0^2	κ_0^4	κ_4^4	κ_0^6	κ_4^6
	$\sqrt{2}$	$2\sqrt{2}$	$2\sqrt{2}$	4	4
	A_0^2	A_0^4	A_4^4	A_0^6	A_4^6
	493.5 meV	179.0 meV	12.3 meV	63.0 meV	5.4 meV
PCEM modified	493.5 meV	179.0 meV	**105.3 meV**	63.0 meV	5.4 meV

Supplementary Table S4 | Crystal field parameters computed from the PCEM models.

The uniaxial terms $A_0^k\, C_0^k(\theta,\varphi)$ commute with the z–projected total moment operator $\hat{J}_z$, therefore they preserve the corresponding quantum number m_j . Conversely, the transverse terms $A_4^k\, C_4^k(\theta,\varphi)$ mix states separated by $\Delta m_j = 4$ and open underbarrier spin-flip transitions accessible with tunnelling electrons.

The zero-field splitting of the lowest atomic multiplet for a Dy in $4f^9 6s^2$ configuration is shown in Fig. S9a, together with the comparison with calculations of the Dy in $4f^{10} 6s^2$ configuration using the same PCEM model, see Fig. S9b. The additional electron in the 4f orbitals reduces the asphericity of the 4f charge and, consequently, the energy separation of the magnetic levels[20,21]. The large difference in the expected barrier height of the two configurations allows us to infer about the $4f^9$ occupation of the

Dy atoms on MgO/Ag(100). The larger charge anisotropy of the $4f^9$ configuration also accounts for the difference in anisotropy barrier between Dy and Ho single atom magnets[1,12,22].

The value of the highest energy states in the $J = 15/2$ multiplet calculated from the bare PCEM model matches well with the energy of highest switching threshold observed in our measurements (see Fig. 1c). However, as it will be discussed in the following, the calculated switching rates are much lower than the experimental values (See also Fig. S10). In order to increase the rate of switching, we increase the contribution of the mixing term A_4^4 until reaching a satisfying agreement with the experiment. The corresponding CF parameters for this modified PCEM configuration is shown in Table S3. This additional mixing does not significantly alter the splitting of the states (see Fig. S8).

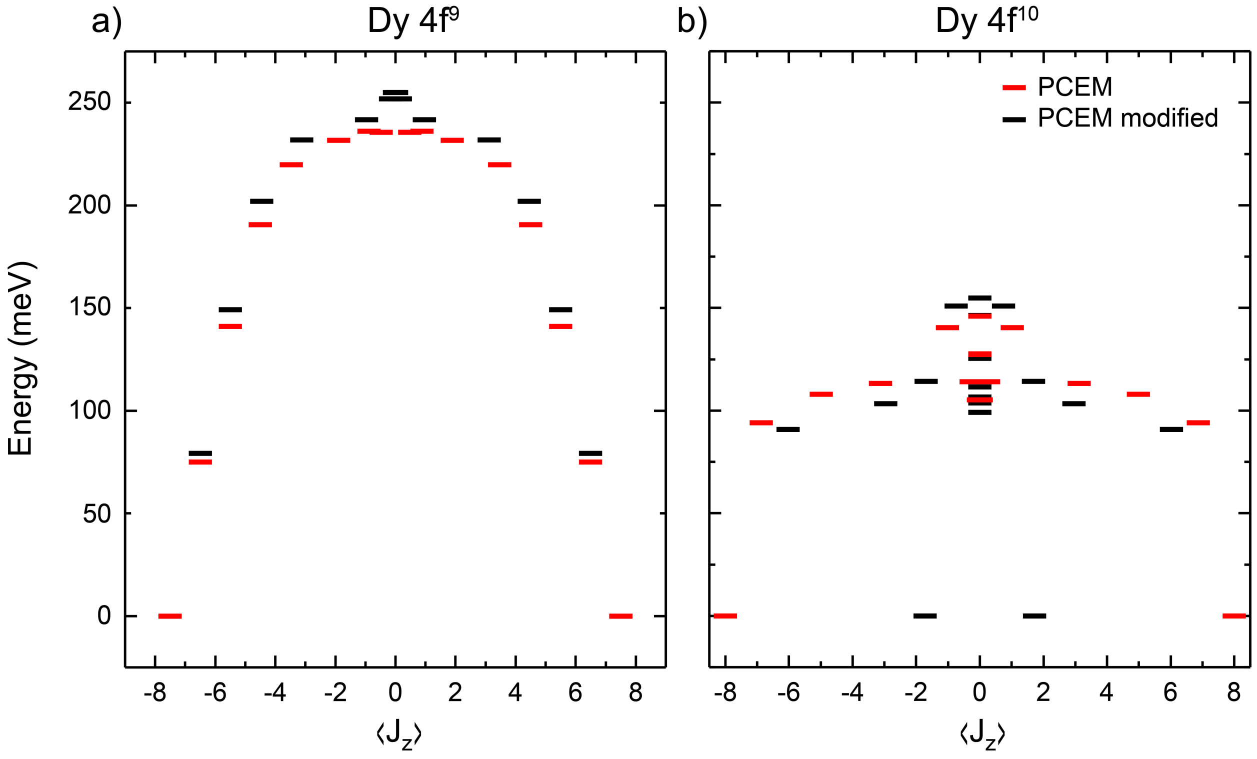

Figure S8 | Zero-field splitting of the Dy atoms. (a) Magnetic levels of Dy in the [Xe]$4f^9 6s^2$ electronic configuration calculated using both bare and modifies PCEM model (b) Comparison with the magnetic levels of Dy in the [Xe]$4f^{10} 6s^2$ configuration.

The voltage-dependent switching rate of Dy is evaluated from the multiplet model by calculating the probability of inducing a reversal via spin–excitation events. We consider reversal paths involving an excitation towards an intermediate state with a single electron scattering process, and a successive decay to a spin state that has opposite orientation with respect to the initial one. The probability of a reversal path that starts from the initial state $i_{\uparrow}$ through an intermediate state j ending in any final state $f_{\downarrow}$ with energy $E_f < E_j$ is calculated as the product of the two spin-excitation events:

$$P_{rev}(j) = P(i_{\uparrow} \rightarrow j) \times \sum_{f \in \{\downarrow, E_f < E_j\}} P(j \rightarrow f_{\downarrow})$$

where $P(i_{\uparrow} \rightarrow j) = \left[\langle i|\hat{\boldsymbol{\sigma}} \cdot \hat{\boldsymbol{J}}|j\rangle\right]^2 / J(J+1)$, with $\boldsymbol{\sigma}$ being the spin of the tunnelling electrons and $\hat{\boldsymbol{\sigma}} \cdot \hat{\boldsymbol{J}} = \hat{J}_z\hat{\sigma}_z + \frac{1}{2}\left(\hat{J}_+\hat{\sigma}_- + \hat{J}_-\hat{\sigma}_+\right)$ is the transition operator related to the scattering with a tunnelling electron[23]. The main reversal mechanisms can be separated into three categories based on the change in the quantum angular momentum operator during the excitation process, with $\Delta m = 0$ transitions associated to the $\hat{J}_z\hat{\sigma}_z$ operator, and $\Delta m = \pm 1$ associated to the $\hat{J}_\pm\hat{\sigma}_\mp$ ladder operators (see Fig. 1c and d).

The thresholds E_j required to reach the intermediate state j, the probabilities $P(j)$ for these transitions and the change in angular momentum during the transition are shown in Table S5.

E_j (meV)	$P(j)$	c_j (s^{-1})	Δm
146	5.62×10^{-9}	2.4	+1
194	6.61×10^{-8}	27.9	0
218	1.64×10^{-7}	69.4	0
220	1.01×10^{-7}	42.6	-1
226	1.40×10^{-7}	59.1	+1
245	3.73×10^{-7}	157.4	0
248	1.14×10^{-6}	482.9	+1

Supplementary Table S5 | Switching rates obtained from multiplet calculations. Values of energy thresholds E_j, transition probabilities $P(j)$, switching rates $c_j = c_0 * (I/e) * P(j)$, and variation of the angular momentum Δm calculated for the modified PCEM model. For the switching rates, we used $c_0 = 0.045$ and $I = 1.5$ nA.

The Dy atoms on MgO/Ag(100) being Kramer's ions with a ground state $< J_Z > = \pm 15/2$, they should be protected against quantum tunnelling of the magnetization (QTM) by time reversal symmetry. Due to this, the tunnel splitting between the doubly degenerate levels should be exactly zero. In real systems, however, the presence of transverse magnetic perturbations such as the dipolar field from the neighbouring spins as well as non-axial components of the tip-field, may lead to a finite QTM rate if the ground state doublet is not perfectly axial. To verify the axiality of the doubly-degenerate levels in the Dy atoms, we calculate the values of parallel (g_z) and transverse (g_x) g-factors, as it is also commonly done for molecular magnets[20]. Our multiplet analysis shows that the doublet is essentially axial with g_z = 19.7919 and g_x = $1.9 * 10^{-6}$. This value of g_x compares very well with the value reported by Guo et al.[24], where a value of g_x = 0 is reported up to the 5th decimal. Similar to that case, Dy atoms on MgO should also be very insensitive to any transverse field, and hence quantum tunnelling should be essentially suppressed. In line with this calculation, we note that all attempts to see switching in the Dy atoms while sweeping the magnetic field across zero have failed. This includes measurements at vanishing magnetic fields for 10 Fe-Dy pairs including those shown in Fig. 3a, the four structures shown in Fig. 4a, as well as the Fe-Dy_4 and Fe-Dy_3 shown in Fig. 4b and 4d. Nearly 100 sweeps on each of these structures within 30 mT never exhibited any switching of the Dy magnetic orientation.

KD	g_x	g_z
2	1.8751797319861e-006	19.791919179346
4	0.0024542465886489	16.8866852253
6	0.0038426437797675	14.065213700788
8	1.4738026540553	11.078514182201
10	0.54160241675803	7.0505239564175
12	10.041247337566	2.0972078608025

14	9.580458229705	0.21734299992859
16	10.011784880366	0.4330854679717

Supplementary Table S6 | Transverse (x) and parallel (z) g-factors as obtained from multiplet calculations. The values are reported according to the sequence of Kramer's doublets (KD).

We finally compute the voltage-dependent switching using the piecewise linear function $\Gamma(\mathrm{eV}) = \sum_{i=1}^{2} c_i \cdot (eV - E_i)/E_i$, with the switching rates $c_j = c_0 * (I/e) * P(j)$. The corresponding values are also summarized in Table S5. Here we use $c_0 = 0.045$ as the only fit parameter serving as global pre-factor to match the experimental rates. This pre-factor indicates the fraction of tunnelling electrons that can interact with the magnetic states and potentially participate in the spin-excitation process. This low number suggests that only a few rare tunnelling events that affect the 4f orbitals are effective in the reversal of the Dy spin. The results of the computation using both bare and modified PCEM model of the crystal field are shown in Fig. S9. As previously anticipated, the results obtained using the bare PCEM model, are in poor agreement with the experiment. Only by increasing the transverse A_4^4 term of the CF field it is possible to achieve a satisfying match with the data. The need of increasing the four-fold symmetric terms suggests that the level composition cannot be fully reproduced by only considering the electrostatic interaction with the surrounding Mg and O ions. We speculate that the origin of this additional term originate from the deformation of the outer 6s orbitals due to the hybridization with the neighbouring Mg atoms[16], which is expected to provide an additional non-spherical contribution to the energy of the 4f electrons not captured in the PCEM model.

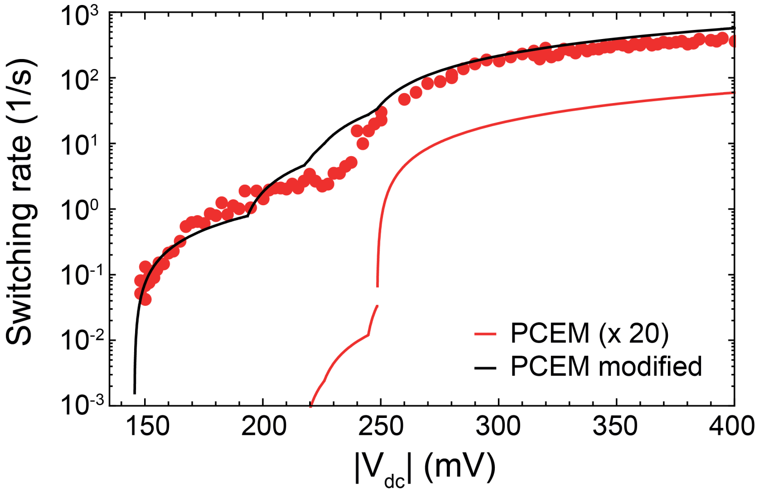

Figure S9 | Switching rates of Dy atoms. Calculated voltage-dependent spin switching for both bare and modified PCEM models. The switching rate of the bare PCEM model is shown magnified by a factor 20 to approach the experimental values.

References

1. Natterer, F. D. *et al.* Reading and writing single-atom magnets. *Nature* **543**, 226–228 (2017).
2. Loth, S., Baumann, S., Lutz, C. P., Eigler, D. M. & Heinrich, A. J. Bistability in Atomic-Scale Antiferromagnets. *Science* **335**, 196–199 (2012).
3. Khajetoorians, A. A. *et al.* Current-Driven Spin Dynamics of Artificially Constructed Quantum Magnets. *Science* **339**, 55–59 (2013).
4. Paul, W. *et al.* Control of the millisecond spin lifetime of an electrically probed atom. *Nat. Phys.* **13**, 403–407 (2016).
5. HONDA, K., KAYA, S. & MASUYAMA, Y. On the Magnetic Properties of Single Crystals of Iron. *Nature* **117**, 753–754 (1926).
6. Halilov, S. V., Perlov, A. Ya., Oppeneer, P. M., Yaresko, A. N. & Antonov, V. N. Magnetocrystalline anisotropy energy in cubic Fe, Co, and Ni: Applicability of local-spin-density theory reexamined. *Phys. Rev. B* **57**, 9557–9560 (1998).
7. Steinbrecher, M. *et al.* Absence of a spin-signature from a single Ho adatom as probed by spin-sensitive tunneling. *Nat. Commun.* **7**, 10454 (2016).
8. Seifert, T. S. *et al.* Longitudinal and transverse electron paramagnetic resonance in a scanning tunneling microscope. *Sci. Adv.* **6**, eabc5511 (2020).

9. Willke, P. *et al.* Tuning Single-Atom Electron Spin Resonance in a Vector Magnetic Field. *Nano Lett.* **19**, 8201–8206 (2019).

10. Reynolds, R. W., Boatner, L. A., Chen, Y. & Abraham, M. M. EPR investigations of Dy3+ and Yb3+ in alkaline-earth oxide single crystals. *J. Chem. Phys.* **60**, 1593–1596 (1974).

11. Fuller, G. H. Nuclear Spins and Moments. *J. Phys. Chem. Ref. Data* **5**, 835–1092 (1976).

12. Choi, T. *et al.* Atomic-scale sensing of the magnetic dipolar field from single atoms. *Nat. Nanotechnol.* **12**, 420–424 (2017).

13. Yang, K. *et al.* Engineering the Eigenstates of Coupled Spin-$1/2$ Atoms on a Surface. *Phys. Rev. Lett.* **119**, 227206 (2017).

14. Haverkort, M. W. Quanty for core level spectroscopy - excitons, resonances and band excitations in time and frequency domain. *J. Phys. Conf. Ser.* **712**, 012001 (2016).

15. Robert D. Cowan. *The Theory of Atomic Structure and Spectra*. (University of California Press, 1981).

16. Singha A. et al. Orbital-resolved single atom magnetism measured with X-ray absorption spectroscopy. *arXiv*.

17. Singha, A. *et al.* 4f occupancy and magnetism of rare-earth atoms adsorbed on metal substrates. *Phys. Rev. B* **96**, 224418 (2017).

18. Görller-Walrand, C. & Binnemans, K. Chapter 155 Rationalization of crystal-field parametrization. in *Handbook on the Physics and Chemistry of Rare Earths* vol. 23 121–283 (Elsevier, 1996).

19. Uldry, A., Vernay, F. & Delley, B. Systematic computation of crystal-field multiplets for x-ray core spectroscopies. *Phys. Rev. B* **85**, 125133 (2012).

20. Rinehart, J. D. & Long, J. R. Exploiting single-ion anisotropy in the design of f-element single-molecule magnets. *Chem. Sci.* **2**, 2078-2085 (2011).

21. Ungur, L. & Chibotaru, L. F. Strategies toward High-Temperature Lanthanide-Based Single-Molecule Magnets. *Inorg. Chem.* **55**, 10043–10056 (2016).

22. Natterer, F. D., Donati, F., Patthey, F. & Brune, H. Thermal and Magnetic-Field Stability of Holmium Single-Atom Magnets. *Phys. Rev. Lett.* **121**, 027201 (2018).

23. Miyamachi, T. *et al.* Stabilizing the magnetic moment of single holmium atoms by symmetry. *Nature* **503**, 242–246 (2013).

24. Guo, F.-S. *et al.* Magnetic hysteresis up to 80 kelvin in a dysprosium metallocene single-molecule magnet. *Science* **362**, 1400-1403 (2018).
